# Supplementary material for: Preferential Localization of MUC1 Glycoprotein in Exosomes Secreted by Non-Small Cell Lung Carcinoma Cells
Source: Int J Mol Sci. 2019 Jan 14;20(2):323. doi: 10.3390/ijms20020323 (PMC6358839; doi:10.3390/ijms20020323)
Supplement: Supplementary file 1 [file ijms-20-00323-s001.zip › ijms-419133 Supplementary files - new/Table S3.docx]

Table S3: Characteristics of 27 NSCLC patients and 16 healthy controls in this study

|  | NSCLC (n=27) | Controls (n=16) |
| --- | --- | --- |
| **Age (median, range)** | 64.6 (42-86) | 57.4 (35-71) |
| **Gender, n (%)** |  |  |
| Male | 14 (51.9%) | 9 (56.3%) |
| Female | 13 (48.1%) | 7 (43.7%) |
| **TNM Stage, n (%)** |  |  |
| Ⅰ | 2 (7.4%) |  |
| Ⅱ | 2 (7.4%) |  |
| Ⅲ | 5 (18.5%) |  |
| Ⅳ | 18 (66.7%) |  |
| **Histology, n (%)** |  |  |
| Adenocarcinoma | 21 (77.8%) |  |
| Squamous carcinoma | 4 (14.8%) |  |
| Others | 2 (7.4%) |  |
| **Smoking, n (%)** |  |  |
| Smoker | 9 (33.3%) |  |
| Nonsmoker | 18 (66.7%) |  |
| **EGFR mutation, n (%)** |  |  |
| Yes | 19 (70.4%) |  |
| No | 8 (29.6%) |  |
