# Supplementary material for: Preferential Localization of MUC1 Glycoprotein in Exosomes Secreted by Non-Small Cell Lung Carcinoma Cells
Source: Int J Mol Sci. 2019 Jan 14;20(2):323. doi: 10.3390/ijms20020323 (PMC6358839; doi:10.3390/ijms20020323)
Supplement: Supplementary file 1 [file ijms-20-00323-s001.zip › Supplementary files/Figure S1 legend.docx]

**Figure S1. Trypsin-digested MUC1 peptide spectrums identified in the exosomes of NCI-H838 cell lines.** (A) Amino acid sequences of trypsin-digested MUC1 peptides; (B,C,D) Spectra of MUC1 peptides identified by LC-MS/MS analysis.
