# Supplementary figures and images for: Preferential Localization of MUC1 Glycoprotein in Exosomes Secreted by Non-Small Cell Lung Carcinoma Cells
Source: Int J Mol Sci. 2019 Jan 14;20(2):323. doi: 10.3390/ijms20020323 (PMC6358839; doi:10.3390/ijms20020323)

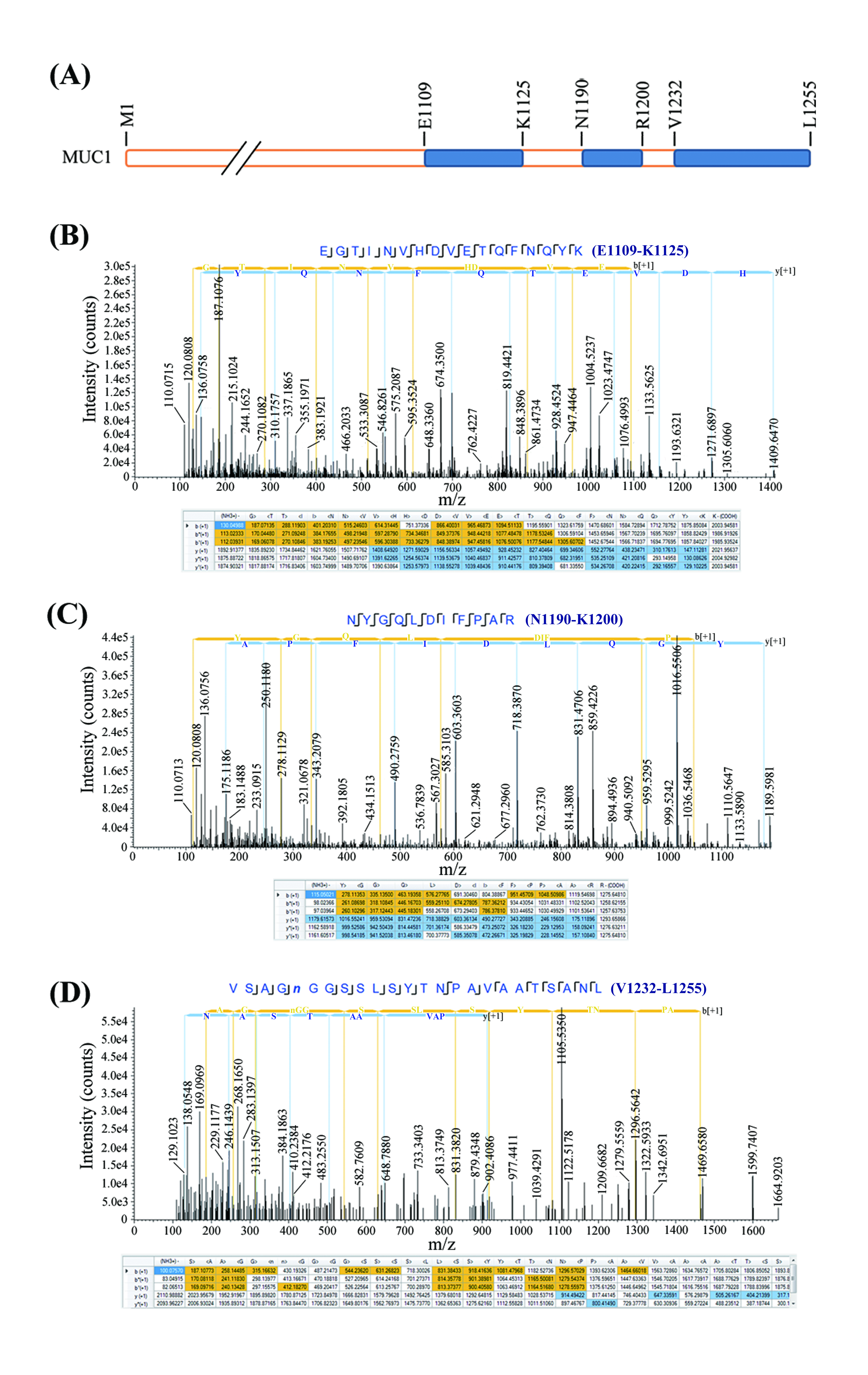

Supplement: Supplementary file 1 [file ijms-20-00323-s001.zip › Supplementary files/Figure S1.tif]
